# Supplementary material for: The WRKY28-BRC1 Transcription Factor Module Controls Shoot Branching in Brassica napus
Source: Plants (Basel). 2025 Feb 6;14(3):486. doi: 10.3390/plants14030486 (PMC11820759; doi:10.3390/plants14030486)
Supplement: Supplementary file 1 [file plants-14-00486-s001.zip › Supplementary Figures.pdf]

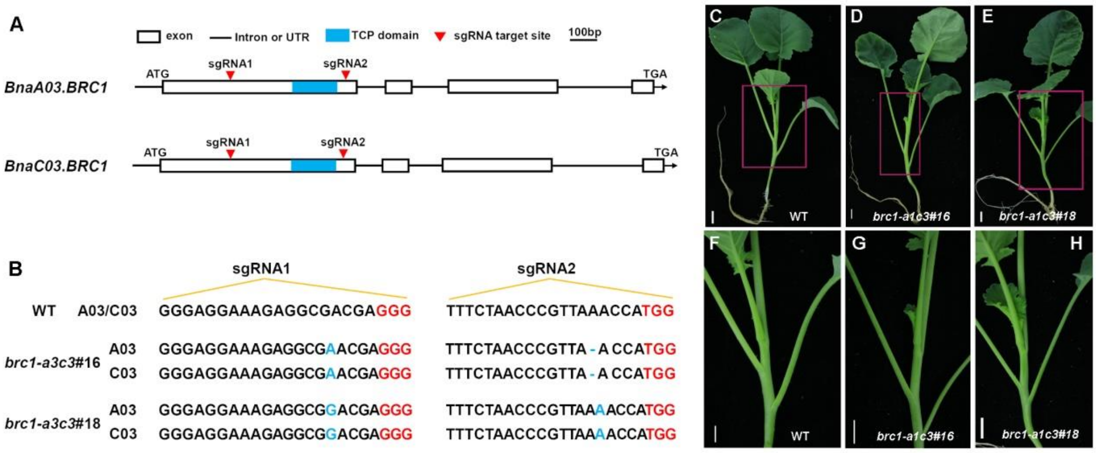

Figure S1. The *brc1-a3c3* lines no significant differences from WT. (A) Schematic diagram to show the sequence characterization of *BnaA03.BRC1* and *BnaC03.BRC1* and the target sites of sgRNAs using the CRISPR/Cas9 system. ATG, start codon; TGA, stop codon. (B) The edit types of two independent homozygous mutants are shown. The protospacer-adjacent motifs (PAM) are marked in red. (C~E) Phenotype of WT and the *brc1-a3c3* lines; ca. six-week-old plants were imaged; scale bar, 1 cm. (F~H) Close-up view of the red square regions in C, D and E; scale bar, 1 cm.

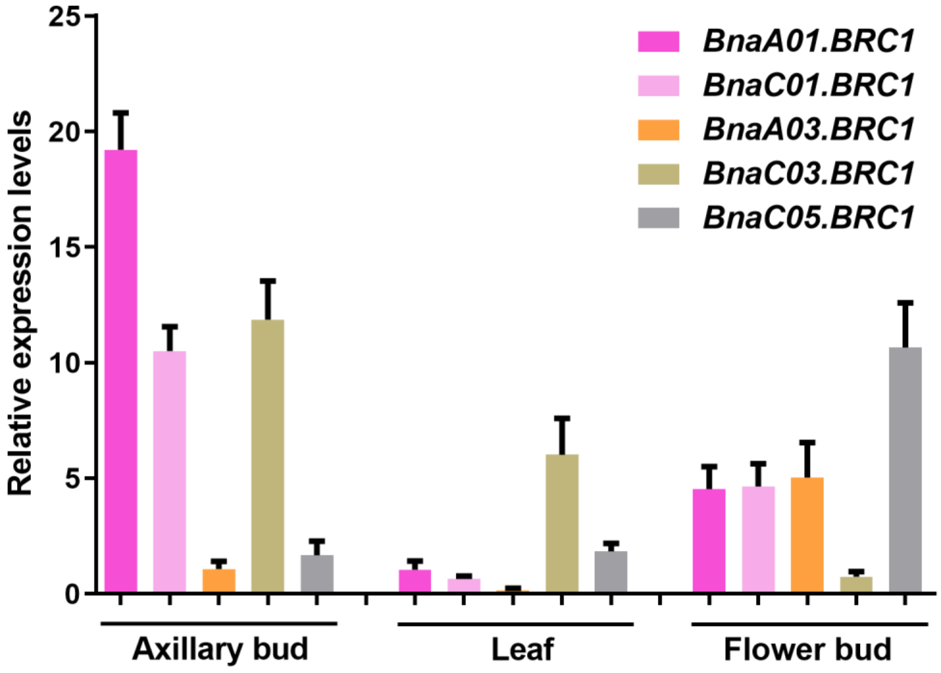

Figure S2. Expression analysis of five *BRC1* copies of rapeseed in axillary buds, leaves and flower buss of WT. The mean values of *BnaA01.BRC1* in leaf were used to normalize the expression levels.

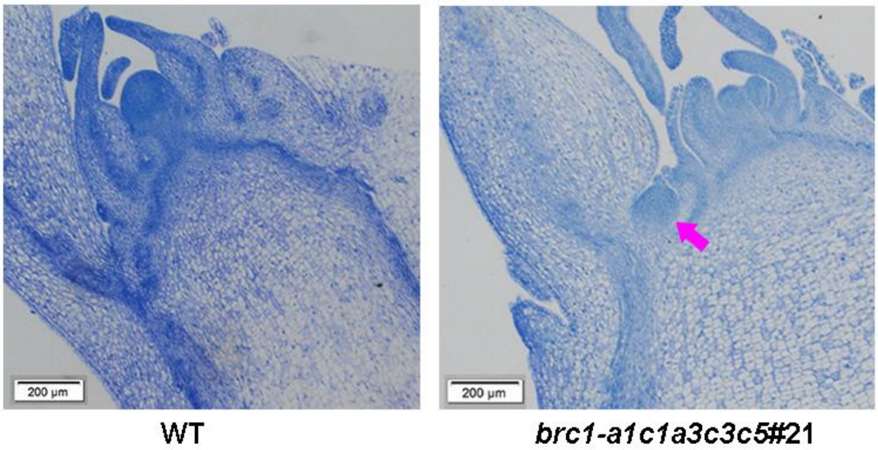

Figure S3. Paraffin sections of the shoot apical meristem of WT and the typical *brc1* mutant. Outgrowthed axillary bud is indicated by magenta arrows.

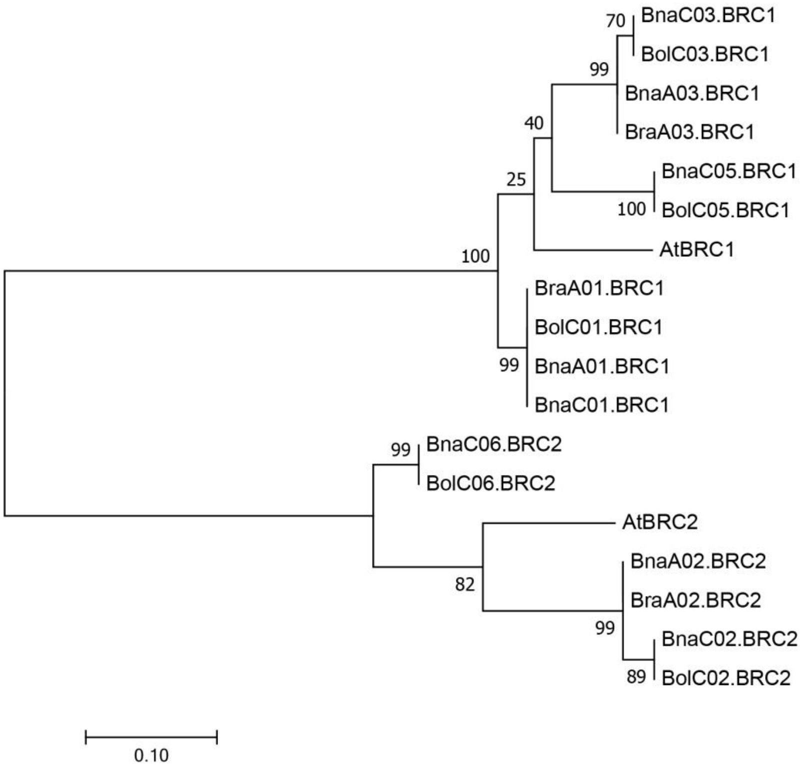

Figure S4. Phylogenetic analysis showing the relationship between each copy of BRC1/BRC2 in *Brassica* species and *Arabidopsis*.

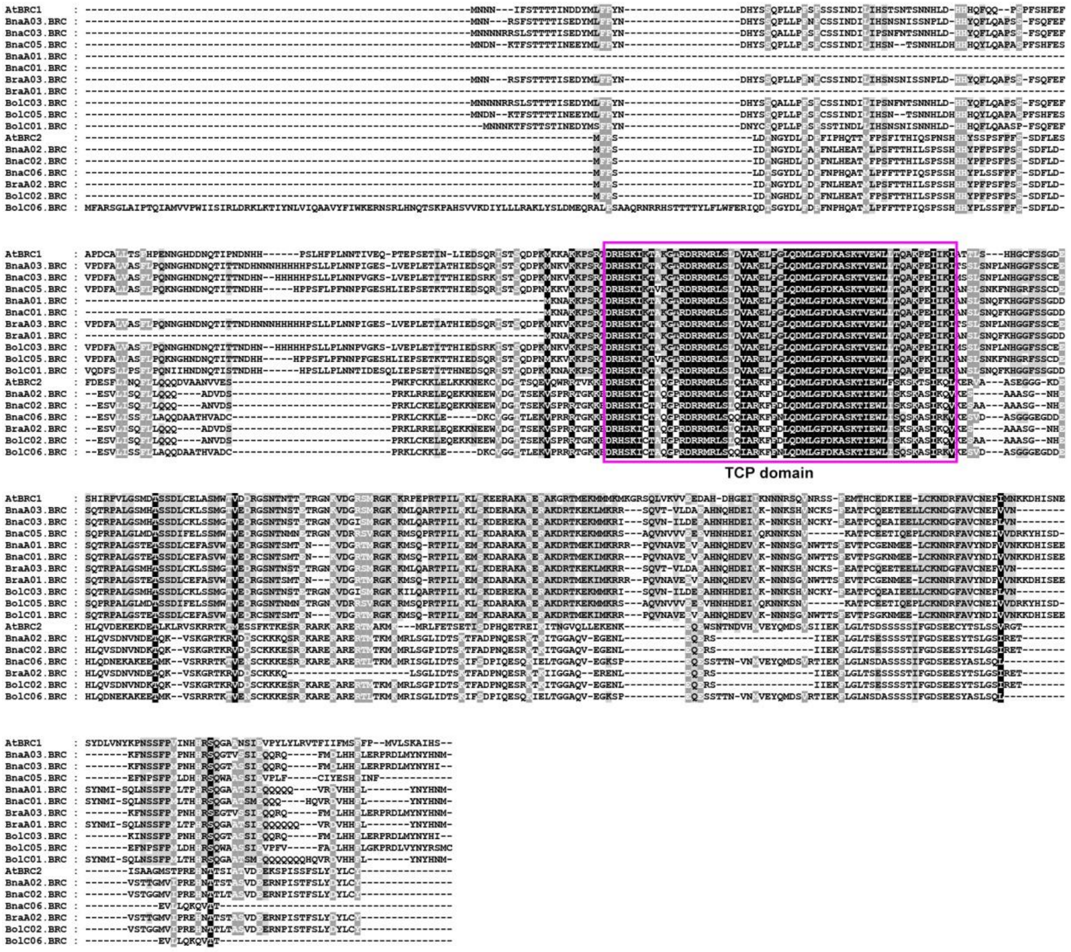

Figure S5. Sequence alignment of identified BRC1/BRC2 copies in *Brassica* species and *Arabidopsis*. Amino acid sequences were used for alignment, and the identical amino acids and similar amino acids were displayed with black and grey shading respectively. The TCP domain was marked with magenta horizontal line.

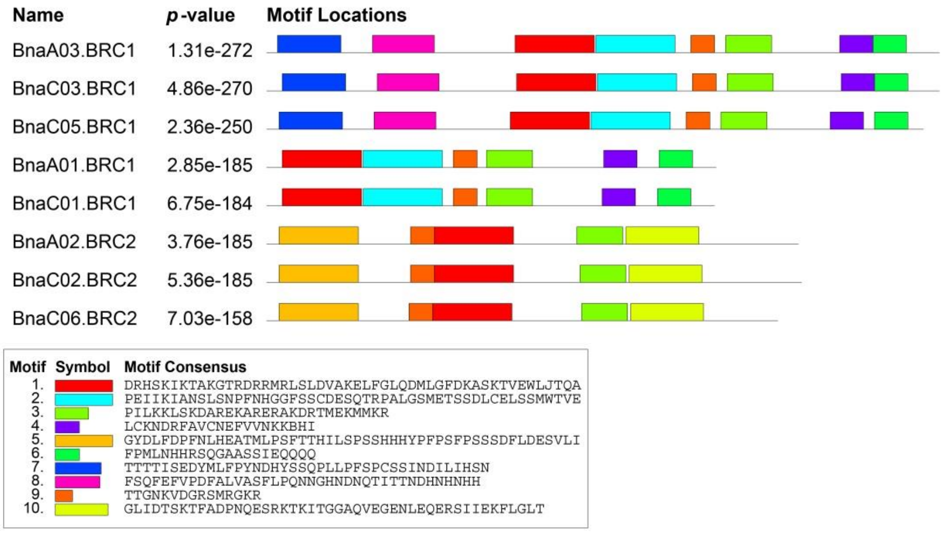

Figure S6. Conserved motifs of five BRC1 copies and three BRC2 copies were predicted by MEME.
